# Safety and efficacy of subcutaneous and continuous intravenous infusion rIL-2 in patients with metastatic renal cell carcinoma

**DOI:** 10.1038/sj.bjc.6601709

**Published:** 2004-03-02

**Authors:** P F Geertsen, M E Gore, S Negrier, J M Tourani, H von der Maase

**Affiliations:** 1 Department of Oncology, University of Copenhagen in Herlev Hospital, Amtssygehuset i Herlev, Denmark; 2 Medical Oncology, The Royal Marsden Hospital NHS Trust, Fulham Road, London, UK; 3 Centre Léon Bérard, 28 Rue Laennec, Lyon, France; 4 Department of Medical Oncology, CHU de Poitiers, Rue de la Milétrie, Poitiers Cedex 86021, France; 5 Department of Oncology, Aarhus University Hospital, Nørrebrogade 44, Aarhus C, Denmark

**Keywords:** interleukin-2, metastatic renal cell carcinoma, subcutaneous administration, continuous intravenous infusion, safety, efficacy

## Abstract

A retrospective analysis was conducted on data from four open-label, nonrandomised, phase II trials of recombinant interleukin-2 (rIL-2) in patients with metastatic renal cell carcinoma to compare the safety and efficacy of administration by subcutaneous (s.c.) and continuous intravenous (c.i.v.) infusion (*n*=103 s.c. and *n*=225 c.i.v.). No statistically significant differences were found between the cohorts in terms of overall response rate (s.c.: 13.6% *vs* c.i.v.: 12.4%, *P*=0.77), response duration (s.c.: 9.8 months *vs* c.i.v.: 10.1 months, *P*=0.99), and overall survival (*P*=0.08). Compared with c.i.v. administration, more patients in the s.c. cohort experienced stable disease (50.5 *vs* 29.8%) and fewer underwent disease progression (35.0 *vs* 43.6%). Subcutaneous administration was associated with a significantly lower incidence of grade 3 or 4 adverse events (46 *vs* 76%; *P*<0.001), and fewer s.c. patients required dose reductions because of toxicity (20 *vs* 82%). At the doses and within the schedules tested, this comparative analysis did not detect any difference in efficacy between s.c. and c.i.v. administration of rIL-2 in terms of overall survival, duration of response and response rate in patients with metastatic renal cell carcinoma. However, s.c. delivery of rIL-2 was associated with improved tolerability.

Metastatic renal cell carcinoma (mRCC) carries a poor prognosis. The response rate to chemotherapy is low and responses are of short duration (Heinzer *et al*, 2001). Immunotherapeutic agents are more successful, and studies with recombinant human interleukin-2 (rIL-2), the first cytokine registered for this indication, have demonstrated survival benefits over chemotherapy for the majority of patients with mRCC (Jones *et al*, 1993; Bordin *et al*, 2000; Fisher *et al*, 2000; Atzpodien *et al*, 2002; Lissoni *et al*, 2002; Pantuck *et al*, 2002).

The clinical development of rIL-2 over the past 20 years has involved investigation of its clinical effects in several regimens using different routes of administration. In initial studies by Rosenberg and coworkers at the National Cancer Institute in the United States, rIL-2 was administered as high-dose intravenous bolus (i.v.b.) injection (Rosenberg *et al*, 1985). A typical i.v.b. regimen comprises the repeated administration of 600 000 IU kg^−1^ of rIL-2 over a period of several days (Taneja *et al*, 1995). Data from these i.v.b. studies showed that this regimen has efficacy in terms of regression in several types of cancer, particularly renal cell carcinoma and benefits in long-term survival have been reported (Fisher *et al*, 2000). However, the risk of severe adverse events with high-dose bolus rIL-2 treatment necessitates patients to be hospitalised for drug administration and also limits the utility of the cytokine to the minority of patients who have good performance status (Redman and Chang, 2000).

Further research showed that the tolerability of rIL-2 could be improved without compromising efficacy by delivering intermediate doses by continuous intravenous infusion (c.i.v.) (West *et al*, 1987). The introduction of short treatment interruptions in this regimen improved the toxicity profile compared with i.v.b. delivery.

To facilitate outpatient treatment, and to enable rIL-2 to be accessible to more patients, recent studies have focused on further improving the tolerability of IL-2 regimens. Low-dose subcutaneous (s.c.) regimens, for example 9–18 million IU rIL-2 delivered daily for several weeks, have been investigated in many studies (Buter *et al*, 1993; Taneja *et al*, 1995; Tourani *et al*, 1996). Findings from these studies have shown that the s.c. administration of rIL-2 is both efficacious and has an improved side-effect profile over c.i.v. delivery.

Subcutaneous regimens with rIL-2 alone or in combination with other agents have been studied intensively in mRCC (Palmer *et al*, 1993; Tourani *et al*, 1998; Negrier *et al*, 2000; Buzio *et al*, 2001; Gez *et al*, 2002). A review of data from several single-agent trials found no difference in overall response rates and complete response rates between bolus, c.i.v., and s.c., although s.c. IL-2 showed less toxicity than i.v. administration (Bukowski, 1997).

In the absence of large controlled trials comparing the clinical effects of delivering IL-2 by c.i.v. or s.c., we conducted this retrospective study. Pooled data from four open-label, nonrandomised phase II studies were evaluated to assess s.c. and c.i.v. rIL-2 routes of administration in terms of safety and efficacy.

## MATERIALS AND METHODS

### Study design

This retrospective analysis included data from four open-label, nonrandomised, multicentre, phase II studies. Two of the studies used s.c. rIL-2 (study SC1 [Protocol NL-MP-100 {Sleijfer *et al*, 1992; Buter *et al*, 1993}]; study SC2 [Protocol EC-MP-101 {Tourani *et al*, 1996}]) and two used c.i.v. rIL-2 (study CIV1 [Protocol EC-L2-008 {von der Maase *et al*, 1991; Geertsen *et al*, 1992; Negrier *et al*, 1992}] and study CIV2 [Protocol EC-MP-001 {Gore *et al*, 1994}]).

### Patients

The analysis comprised pooled data from 103 patients with mRCC, included in two rIL-2 s.c. studies, and 225 patients with mRCC, included in two rIL-2 c.i.v. studies. Before study entry, the disease stage of each patient was determined by full clinical examination. Patients with histological proven, clinical measurable mRCC, and with an Eastern Cooperative Oncology Group (ECOG) performance status 0 and 1 for the c.i.v. cohort and 0, 1, and 2 for the s.c. cohort, were eligible. Histological subtype (e.g. clear cell type, papillary type) was not prospectively defined as a stratification factor. All trials excluded patients with brain metastases, secondary neoplasms, performance status >2, or those who had been previously treated with rIL-2. Only the s.c. trials permitted inclusion of patients with concomitant illnesses, for example those with cardiovascular disease (previous myocardial infarction, angina pectoris, arrhythmias, cardiac ischaemia, aortic-femoral bypass, valvular disease), spinal cord lesion, bilateral nephrectomy, transient ischaemic attacks, previous cerebrovascular accident, and porphyria.

### Treatments

**Figure 1:**
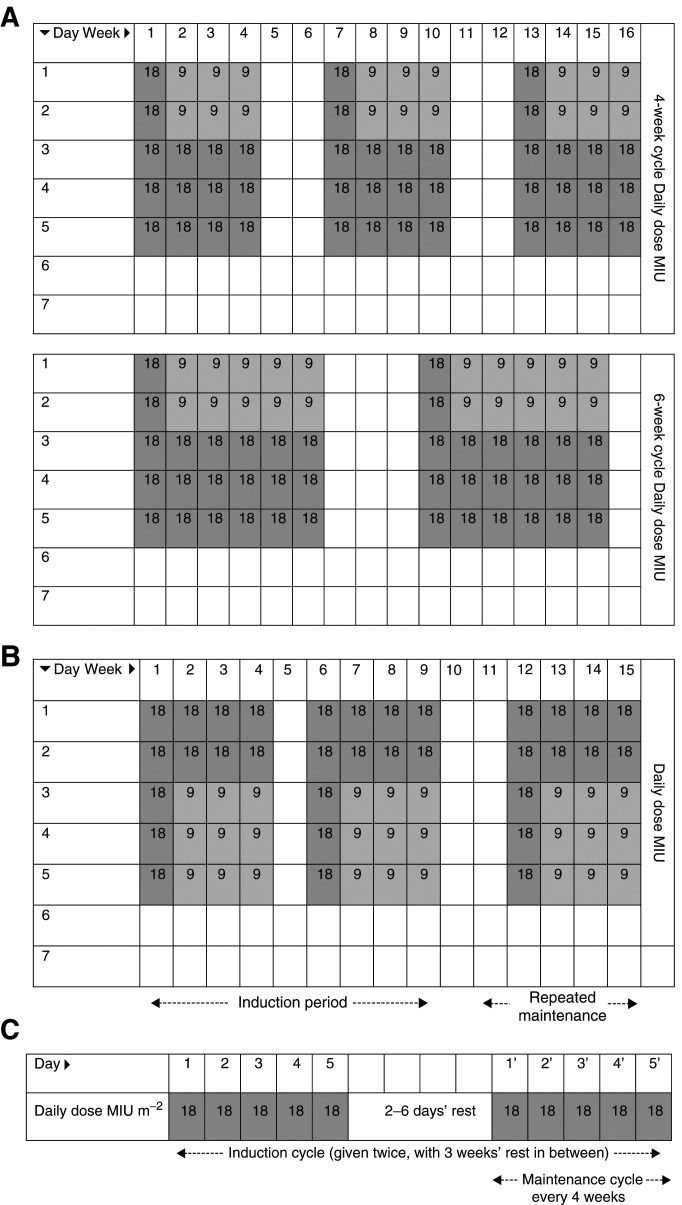
Dosing regimens used in studies analysed in this retrospective analysis (**A**) Study SC1 (12-week subcutaneous treatment using 4- or 6-week cycles – Protocol NL-MP-100). (**B**) Study SC2 (subcutaneous treatment until disease progression/unacceptable toxicity – Protocol EC-MP-101). (**C**) Studies CIV1 and CIV2 (continuous intravenous treatment until disease progression or unacceptable toxicity or up to a maximum of four maintenance cycles – Protocols EC-L2-008 and EC-MP-001).

All patients were treated with commercial rIL-2 (Proleukin®: aldesleukin: modified recombinant Human Interleukin-2), purchased from Chiron B.V. (Amsterdam). Continuous intravenous infusions were administered in oncology wards with close monitoring during therapy. Subcutaneous injection was administered at home or in an outpatient clinic. The dosing regimens used in these studies are illustrated in Figure 1

. To make a comparison between s.c. and c.i.v. regimens possible, equivalent c.i.v. dosages (expressed as MIU m^−2^) are given in parentheses after the s.c. dosages. These theoretical equivalents are based on a 68 kg patient, height 173 cm and a calculated body surface area of 1.8 m^2^. Thus, a s.c. dose of 18 MIU day^−1^ would be equivalent to a c.i.v. dose of 10 MIU m^−2^ day^−1^.

#### Subcutaneous cohorts

In study SC1 (Sleijfer *et al*, 1992; Buter *et al*, 1993), s.c. injections were given for a total of 12 weeks, in three 4-week or two 6-week treatment cycles, separated by 2- or 3-week rest periods, respectively (Figure 1A). Patients received 90 MIU (theoretically equivalent to 50 MIU m^−2^ week^−1^) during week 1 of each cycle (18 MIU day^−1^ on days 1–5) and 72 MIU (theoretically equivalent to 40 MIU m^−2^ week^−1^) during weeks 2–4 or 2–6 of each cycle (9 MIU day^−1^ on days 1 and 2 and 18 MIU day^−1^ on days 3–5). The first 29 patients enroled received 6-week treatment cycles; the remainder received 4-week cycles.

In study SC2 (Tourani *et al*, 1996), patients received s.c. rIL-2 induction treatment during weeks 1–4 and 6–9. After a 2-week rest period, responding patients received 4-week maintenance cycles separated by 2-week rest periods (Figure 1B). Induction doses of rIL-2 were 90 MIU (theoretically equivalent to 50 MIU m^−2^ week^−1^) during weeks 1 and 6 (18 MIU day^−1^ on days 1–5), and 63 MIU (theoretically equivalent to 35 MIU m^−2^ week^−1^) during weeks 2–4 and 7–9 (18 MIU day^−1^ on days 1 and 2, and 9 MIU day^−1^ on days 3–5). Maintenance doses were 90 MIU (theoretically equivalent to 50 MIU m^−2^ week^−1^) during week 1 (18 MIU day^−1^ on days 1–5), and 63 MIU (theoretically equivalent to 35 MIU m^−2^ week^−1^) during weeks 2–4 (18 MIU day^−1^ on days 1 and 2, and 9 MIU day^−1^ on days 3–5). Oral acetaminophen (250–500 mg every 4–6 h) was administered concomitantly to prevent pyretic reactions.

#### Continuous intravenous infusion cohorts

For CV1 (Negrier *et al*, 1989; von der Maase *et al*, 1991; Geertsen *et al*, 1992) and CV2 (Gore *et al*, 1994) the same schedule was used. Treatment consisted of two induction cycles (each consisting of two, 5-day dosing periods separated by 2–6 day rest periods) and four maintenance cycles (5-day dosing periods every 4 weeks). Daily doses of 18 MIU m^−2^ were administered by 24-h c.i.v. for 5 days (Figure 1C).

Dose modification was used to control toxicity. Infusion of rIL-2 was interrupted if any of the following complications occurred: hypotension (grade 3 or 4), significant arrhythmia, suspicion of myocardial ischaemia, agitation or persistent confusion, elevation of bilirubin above 5 mg 100 ml^−1^, elevation of serum creatinine above 4.5 mg 100 ml^−1^, bacterial sepsis, or dyspnoea at rest. The dose of rIL-2 was reduced by 50% if any of the following complications occurred during the previous cycle: hypotension (grade 3 or 4), elevation of bilirubin above 6.0 mg 100 ml^−1^, elevation of serum creatinine above 5 mg 100 ml^−1^, and neurotoxicity (of greater severity than grade 3).

### Evaluation of efficacy and safety

Tumour volume was evaluated clinically and radiologically after the induction period and during maintenance periods of treatment. Response to therapy was assessed after each treatment cycle and patients receiving at least one cycle of therapy were evaluable for response. World Health Organization (WHO) criteria (Miller *et al*, 1981) were used to evaluate the tumour response, that is, complete response (CR), partial response (PR), stable disease (SD), progressive disease (PD), overall response rate (number of CRs and PRs), response duration, and for assessing the severity of adverse events. Adverse events were assessed continually throughout the study. Overall survival was measured from the initiation of rIL-2 therapy to the date last known alive or the date of death.

### Statistical analyses

All patients included in the original separate analyses of the four studies were included in this retrospective analysis. The s.c. and c.i.v. treatment cohorts were compared with respect to pretreatment patient characteristics, including potential prognostic factors, using the χ^2^-test for categorical variables and the Wilcoxon rank-sum test for continuous variables. All patients who received at least one dose of rIL-2 were entered and included in this analysis. Overall response to treatment (CR+PR) and overall incidence of adverse events in individual body systems were compared using the χ^2^-test, or Fisher's exact test when the former was inappropriate. Percentages and 95% confidence intervals were calculated for CR, PR, and CR+PR using the Clopper–Pearson formula for binomial distributions. The Kaplan–Meier product-limit method was used to estimate the survival distribution and response duration, which were compared between groups using the log-rank test. To adjust for imbalances in potential baseline prognostic factors for mRCC (including: performance status, prior therapy, prior nephrectomy, number of metastatic sites (1, 2, and ⩾3) and time from diagnosis to treatment (>24 *vs* ⩽24 months) (Palmer *et al*, 1992), and age, a multivariate regression analysis (Cox proportional hazards model) was used to assess survival. All statistical analyses were performed using SAS® version 6.07 or higher (SAS Institute, Cary, NC, USA). Statistical significance was assessed at an *á* level of 0.05 and all reported *P*-values are two-sided.

## RESULTS

### Patients

Baseline characteristics of the patient cohorts treated with s. c. and c.i.v. rIL-2 are shown in Table 1

**Table 1:** Summary of patient characteristics at baseline

	**rIL-2 regimen**			
**Parameter**	**s.c. (*n*=103)**	**c.i.v. (*n*=225)**	**All (*n*=328)**	***P-*value**
*Age (years)*				0.02
Median (range)	59.0 (21–84)	56.0 (20–80)	57.0 (20–84)	

*Sex, N* (%)				ns
Female	34 (33)	69 (31)	103 (31)	
Male	69 (67)	156 (69)	225 (69)	
	
*Weight (kg)*	(*n*=95)	(*n*=222)	(*n*=317)	0.03
Median (range)	74.0 (46–110)	71.0 (41.5–107)	71.0 (41.5–110)	
	
*ECOG performance status, n* (%)				<0.001
0	58 (56)	94 (42)	152 (46)	
1	38 (37)	131 (58)	169 (52)	
2	7 (7)	0 (0)	7 (2)	

*Time from diagnosis to start of treatment, n* (%)				ns
⩽24 months	81 (79)	179 (80)	260 (79)	
>24 months	22 (21)	46 (20)	68 (21)	

*Time from diagnosis to detection of metastasis, n* (%)				ns
⩽24 months	83 (81)	184 (82)	267 (81)	
>24 months	17 (17)	37 (16)	54 (16)	
Unknown	3 (3)	4 (2)	7 (2)	

*Time from detection of metastasis to start of treatment, n* (%)				ns
⩽24 months	94 (91)	214 (95)	308 (94)	
>24 months	6 (6)	7 (3)	13 (4)	
Unknown	3 (3)	4 (2)	7 (2)	

*Prior therapy, n* (%)				
At least one type of prior therapy (excl. surgery)	22 (21)	82 (36)	104 (32)	< 0.01
Surgery	83 (81)	184 (82)	267 (81)	ns
Nephrectomy	87 (76)	173 (77)	251 (77)	ns
Chemotherapy	7 (7)	11 (5)	18 (5)	ns
Radiotherapy	15 (15)	40 (18)	55 (17)	ns
Hormontherapy	0 (0)	43 (19)	43 (13)	<0.001
Immunotherapy	7 (7)	16 (7)	23 (7)	ns

*Metastatic disease at nephrectomy*	(*n*=74)	(*n*=171)	(*n*=245)	ns
Yes	10 (14)	22 (13)	32(13)	

*No. of metastatic sites*	(*n*=101)	(*n*=225)	(*n*=326)	0.04
1	41 (41)	75 (33)	116 (36)	
2	43 (43)	82 (36)	125 (38)	
⩾3	17 (17)	68 (30)	85 (26)	

s.c.=subcutaneous – Study SC1 (Protocol NL-MP-100 [Sleijfer *et al*, 1992; Buter *et al*, 1993]) and Study SC2 (Protocol EC–MP–101 [Tourani *et al*, 1996]).

c.i.v.=continuous intravenous infusion – Study CV1 (Protocol EC-L2–008 [von der Maase *et al*, 1991; Geertsen *et al*, 1992; Negrier *et al*, 1992]) and Study CV2 (Protocol EC-MP-001 [Gore *et al*, 1994]).

ECOG=Eastern Cooperative Oncology Group.

. There were statistically significant differences between groups with respect to age, weight, performance status, number of metastatic sites, patients who had received prior hormone therapy, and patients who had received any type of prior therapy excluding surgery. In general, patients treated with s.c. rIL-2 were older and heavier, had better performance status and fewer metastatic sites, and had received less of at least one type of prior therapy except for surgery than the cohort who received c.i.v. therapy. These differences were all statistically significant (all *P*<0.05).

The groups were well matched with respect to gender; time from initial diagnosis to start of treatment; time from initial diagnosis to detection of metastasis; time from detection of metastasis to start of treatment; and frequency of prior surgery, nephrectomy, chemotherapy, or radiotherapy.

### Treatment

Patients in the s.c. and c.i.v. cohorts spent a similar number of days on the study (mean 90 *vs* 88 days, respectively). Both the mean (692 *vs* 590 MIU) and the median (594 *vs* 562 MIU) cumulative doses received were higher in the s.c. than in the c.i.v. cohort (Table 2

**Table 2:** Summary of rIL-2 doses received and treatment duration

	**rIL-2 regimen**	
**Parameter**	**s.c. (*n*=103)**	**c.i.v. (*n*=225)**
Patients with dose reduction	21 (20%)	184 (82%)
Mean number of days in study	90	88
Median number of days in study	70	73
Range of days in study	14–385	1–268
Mean cumulative dose (MIU)	692	590
Median cumulative dose (MIU)	594	562
Dose range	(135–2232)	(60–1710)

s.c.=subcutaneous; c.i.v.=continuous intravenous infusion.

). A substantially lower proportion of patients in the s.c. group (20%) required dose reduction because of toxicity compared with patients receiving c.i.v. treatment (82%) (Table 2).

### Efficacy

Of the 103 patients treated with s.c. rIL-2 that were included in the analysis, 14 (13.6%, 95% CI: 7.6–21.8%) had an overall response, four (3.9%) had a CR, 10 (9.7%) had a PR, 52 (50.5%) had SD, 36 (35.0%) had disease progression PD, and one (1%) was not evaluable. Of the 225 patients treated with c.i.v. rIL-2, 28 patients (12.4%, 95% CI: 8.4–17.5%) had an overall response, seven (3.1%) had a CR, 21 (9.3%) had a PR, 67 (29.8%) had SD, 98 (43.6%) had PD and 32 (14.2%) were not evaluable (Table 3

**Table 3:** Summary of clinical responses

	**rIL-2 regimen**	
**Parameter**	**s.c. (*n*=103) *n* (%)**	**c.i.v. (*n*=225) *n* (%)**
CR	4 (3.9)	7 (3.1)
PR	10 (9.7)	21 (9.3)
CR+PR (95% CI)	14 (13.6, 7.6–21.8)	28 (12.4, 8.4–17.5)
SD	52 (50.5)	67 (29.8)
PD	36 (35.0)	98 (43.6)
NE	1 (1.0)	32 (14.2)
Median response duration (months, range)	9.8 (4–93+)^a^	10.1 (1–95+))^a^

s.c.=subcutaneous; c.i.v.=continuous intravenous infusion.

a s.c.: *n*=14, c.i.v.: *n*=28 for these calculations; SD=stable disease; CR=complete response; PR=partial response; PD=progressive disease; NE=not evaluable.

). The difference in overall response between the two treatment groups was not statistically significant (s.c. 13.6% *vs* c.i.v. 12.4%; *P*=0.77).

Median duration of response was 9.8 months for the s.c. treatment cohort and 10.1 months for the c.i.v. treatment cohort (Table 3); the difference between the two treatment groups was not significant (*P*=0.99). Response durations for the four complete response patients in the s.c. cohort are 27, 64+, 86+, and 93+ months and for the seven CR patients of the c.i.v. cohort 2, 6, 10, 14+, 47+, 76+, and 95+ months.

**Figure 2:**
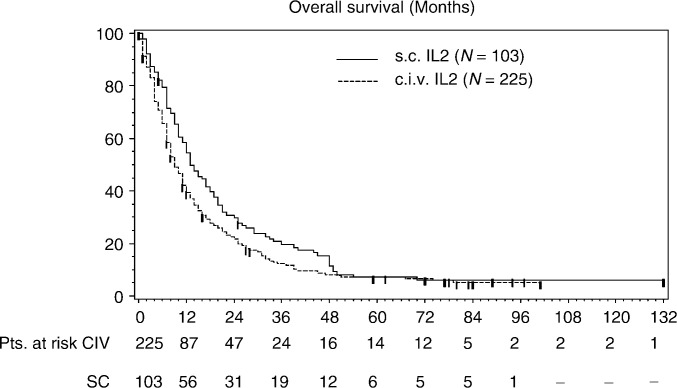
Overall survival in patients with metastatic renal cell carcinoma treated with subcutaneous (s.c.) or continuously infused (c.i.v.) rIL-2; retrospectively pooled data from open-label, nonrandomised trials.

The results of the univariate analysis of overall survival for the two rIL-2 treatment groups revealed a significant survival advantage (*P*=0.03) for the s.c. cohort compared with the c.i.v. cohort. Median survival for the s.c. rIL-2 patients was 13.7 months (95% CI: 10.6–18.1) *vs* 9.1 months (95% CI: 8.1–11.3) for c.i.v. rIL-2. The survival probability over 3 years was higher at each annual time point in the s.c.-treated group than in the c.i.v. cohort. The survival probabilities at 1, 3, and 5 years for the s.c. group were 57, 12, and 8%, respectively. For the c.i.v. groups these were 41, 9, and 8%, respectively (Figure 2

).

The result on the overall survival was based on a retrospective analysis on data of nonrandomised phase II studies. Therefore, a multivariate analysis was done. After correcting for imbalances in baseline characteristics to be potentially prognostic for mRCC, known at the time these studies were done (performance status, number of metastatic sites, time from diagnosis to treatment (Palmer *et al*, 1992), and for significant different baseline characteristics between the two treatment cohorts (age, weight, prior therapy)) the s.c. and c.i.v. regimens were found not to be significant different in terms of overall survival (*P*=0.08).

### Safety

The overall incidence of adverse events was similar in the s.c. and c.i.v. cohorts (*P*=0.66) (Table 4

**Table 4:** Summary of all adverse events, including abnormalities in laboratory values, by body system

	**rIL-2 regimen**				
	**s.c.**		**c.i.v.**		
**Body system**	**(*n*=103)**	**%**	**(*n*=225)**	**%**	***P-*value**
Any adverse event	103	100	222	99	0.66
Body as a whole	99	96	211	94	0.39
Cardiovascular system	19	18	174	77	<0.001
Digestive system	86	83	156	69	0.007
Disease related	0	0	1	<1	1.0
Endocrine system	13	13	2	1	<0.001
Eye	2	2	16	7	0.068
Haemic and lymphatic system	63	61	182	81	<0.001
Metabolic and nutritional disorders	77	75	198	88	0.002
Musculoskeletal system	3	3	6	3	1.0
Mucosa	1	1	23	10	0.002
Nervous system	11	11	67	30	<0.001
Not specified	0	0	1	<1	1.0
Respiratory system	16	16	67	30	0.006
Skin and appendages	41	40	133	59	0.001
Special senses	1	1	0	0	0.31
Urogenital system	4	4	73	32	<0.001
Injection site	52	50	1	<1	<0.001

s.c.=subcutaneous; c.i.v.=continuous intravenous infusion.

). However, comparison of the body systems affected by all adverse events, regardless of their severity, showed that the s.c. and c.i.v. administration routes were associated with significantly different safety profiles (Table 4). Subcutaneous delivery was more frequently associated with adverse events affecting the endocrine system (*P*<0.001). Administration by c.i.v. was more frequently associated with adverse events affecting the cardiovascular, haemic/lymphatic, nervous, and urogenital systems (all *P*<0.001).

Severe adverse events (grade 3 or 4) occurred more frequently in patients who received the c.i.v. regimen than in those treated with s.c. rIL-2 (76 *vs* 46%; *P*<0.001) (Table 5

**Table 5:** Summary of severe (grade 3 or 4) adverse events, including laboratory abnormalities, by body system

	**rIL-2 regimen**				
	**s.c.**		**c.i.v.**		
**Body system**	**(*n*=103)**	**%**	**(*n*=225)**	**%**	***P-*value**
Any adverse event	47	46	171	76	<0.001
Body as a whole	26	25	73	32	0.19
Cardiovascular system	3	3	78	39	<0.001
Digestive system	9	9	30	13	0.23
Endocrine system	1	1	0	0	0.31
Eye	0	0	1	<1	1.0
Haemic and lymphatic system	10	10	22	10	0.98
Metabolic and nutritional disorders	10	10	54	24	0.002
Musculoskeletal system	1	1	2	1	1.0
Nervous system	1	1	28	12	<0.001
Not specified	0	0	1	<1	1.0
Respiratory system	0	0	31	14	<0.001
Skin and appendages	4	4	17	8	0.21
Urogenital system	3	3	24	11	0.018
Injection site	5	5	0	0	1.0

s.c.=subcutaneous; c.i.v.=continuous intravenous infusion.

). More patients in the c.i.v. group, compared with those in the s.c. cohort, experienced severe adverse events of the cardiovascular, respiratory, urogenital systems (all *P*<0.001), and metabolic/nutritional system (*P*=0.002). Individual, severe adverse events that were more commonly reported in the c.i.v. group *vs* the s.c. cohort included fever (23 *vs* 17%), hypotension (36 *vs* 2%), anaemia (9 *vs* 4%), oliguria (6 *vs* 0%), and increased alkaline phosphatase levels (14 *vs* 0%).

Injection site reactions were, as expected, only associated with s.c. administration of rIL-2, with 50% of patients in this group reporting this adverse event. The majority (>95%) of injection site reaction events were graded as mild (grade 1) and usually diminished within 1 week.

Dose modification of rIL-2 was not required by the majority of s.c. patients (80%) but was frequently necessary in patients receiving c.i.v. rIL-2 (82%). Treatment delays were also less frequent in patients in the s.c. cohort than in patients in the c.i.v. group (4 *vs* 20%) (Table 6

**Table 6:** Summary of dose modifications and treatment delays

	**rIL-2 regimen**	
	**s.c.**	**c.i.v.**
	**(*n*=103)**	**(*n*=225)**
*Dose modifications (N/patient)*		
Mean	0.3	6.5
Median	0.0	4.0
Range	0–4	0–49

*Dose not modified* (*N* [%])	82 (80%)	41 (18%)

*Treatment delays* (*N* [%])		
Yes	4 (4%)	45 (20%)
No	99 (96%)	180 (80%)
Unknown	0	18

s.c.=subcutaneous; c.i.v.=continuous intravenous infusion.

).

## DISCUSSION

In this study, we have retrospectively analysed the efficacy and safety of s. c. and c.i.v. routes of administration of rIL-2 in mRCC patients.

The analysis did not detect any significant differences between either method of administration with respect to overall response, median response duration, and overall survival, after correcting for imbalances in baseline prognostic factors (performance status, number of metastatic sites, time from diagnosis to treatment) and baseline characteristics (age, weight, prior therapy). However, a greater proportion of patients in the s.c. cohort experienced SD compared to patients in the c.i.v. group (35.0 *vs* 43.6% respectively). Long-lasting complete responses (>5 years) were found in both cohorts.

Nowadays, histological subtype is a known prognostic factor for renal cell carcinoma. The majority of renal cell carcinoma is of the clear cell carcinoma type (75%). Patients with this subtype benefit most from IL-2 therapy (Wu *et al*, 1998). The outcome of our retrospective analysis could have been influenced by the histological subtypes of the patients. At the time these studies were conducted, histological subtype was not a known prognostic factor for clinical outcome of renal cell carcinoma patients, and therefore no distinction was made prospectively on this parameter. Because of the limitations of our database it is not possible to look back at these specific subtypes. However, clinical trials should include better pathologic stratification at enrolment of RCC patients.

Furthermore, the analysis shows that s.c. administration is associated with an improved tolerability profile, compared with the c.i.v. route of administration. Patients in the s.c. group experienced significantly fewer severe side effects than those in the c.i.v. cohort (46 *vs* 76%, *P*<0.001). Patients in the s.c. cohort also needed fewer toxicity-related dose reductions of rIL-2, compared with those in the c.i.v. group (20 *vs* 82%). This outcome is recently confirmed by Yang *et al* (2003), who compared response rates and overall survival of patients with metastatic renal cell carcinoma in a randomised study receiving either high-dose or one of two low-dose IL-2 regimens (high-dose bolus i.v., low-dose bolus i.v., and s.c. resp.). With a median follow-up of 7.4 years, long-term survival was 21% for all study patients and there were no significant differences in overall survival between the three arms, although the response rate of bolus IL-2 was higher that that of the s.c. IL-2 regimen. However, the safety profile was markedly improved with the s.c. treatment with respect to both i.v. regimens.

However, a notable exception is the significant higher incidence of adverse events concerning the endocrine system found in patients treated by the s.c. route of administration. It is known that reversible thyroid dysfunction occurs in up to 60% of metastatic cancer patients treated with immunotherapy consisting of IL-2 alone or in combination with interferon-*α* or lymphocyte-activated killer cells. It has been associated with favourable tumour response (Weijl *et al*, 1993; Franzke *et al*, 1999). The higher percentage of SD found in patients from the s.c. cohort (50.5 and 29.8% for s.c. and c.i.v. rIL-2 treatment, respectively) may be somehow related by the higher incidence of thyroid dysfunction in this treatment group. It has been speculated that the cytokines enhance the immune response to certain autoantigens as well as to antigens present on tumour cells, or that the immune responses to thyroid tissue and tumour tissue are similarly regulated (Franzke *et al*, 1999).

Our findings have to be considered within the limitations of our analysis. We have compared data from two different cohorts of patients from four different phase II studies, which were conducted at different centres. Although we have adjusted in our statistical analysis for imbalances in prognostic factors known at the time these studies were conducted, they may have biased the results. This may also be valid for other prognostic factors identified afterwards. However, our data are consistent with other studies, suggesting that s.c. administration has similar efficacy as c.i.v. treatment but is associated with milder side effects (Bukowski, 1997).

The improved tolerability of s.c. delivery of rIL-2 may be explained by factors such as the mechanism of action and distribution of rIL-2 within the body. Low doses of rIL-2, delivered by s.c., results in picomolar concentrations of circulating rIL-2 that specifically stimulates production of natural killer cells. In contrast, the higher doses used in c.i.v. administration stimulate expansion of a broad range of immune cells, which are suspected to cause severe adverse events that may occur during treatment (Fehniger *et al*, 2002). Furthermore, s.c. administration of rIL-2 provides a lower and more consistent level of systemic drug exposure than delivery by c.i.v. This also may contribute to the favourable tolerability profile observed with this mode of administration (Konrad *et al*, 1990; Leahy *et al*, 1992; Yang and Rosenberg, 1997).

The improved tolerability of s.c. rIL-2 supports its administration in the outpatient setting. Compared to c.i.v. delivery in hospital, treatment with s.c. rIL-2 at home or in the community may lead to an improvement in patients’ quality of life and reduce healthcare management costs. In addition, the improved tolerability of s.c. delivery may allow rIL-2 to have clinical utility in a wider variety of patients, including those with poor performance and patients with concomitant systemic disease.

In conclusion, this retrospective analysis did not detect any difference in efficacy between s.c. and c.i.v. administration of rIL-2 in terms of overall survival, duration of response and response rate in patients with mRCC. However, s.c. delivery of rIL-2 has improved tolerability compared with c.i.v. administration. These data are consistent with the growing body of evidence that shows s.c. IL-2 to be effective, well tolerated, and suitable for use as an outpatient treatment (Bukowski, 1997). In the absence of results from large controlled trials, it is hoped that data from our analysis will provide additional evidence to assist clinicians' use of rIL-2 in mRCC in their current clinical practice. Other factors, that may well help maximise the effectiveness of rIL-2 therapy, include the identification of new prognostic factors (van Herpen and de Mulder, 2002), the identification of biological markers of immunotherapies' efficacy and combination with other agents (e.g. vaccines, dendritic cells) (Malaguarnera *et al*, 2001). To optimise fully the clinical application of rIL-2, these and other questions need to be investigated so that the most appropriate rIL-2 dose and scheduling regimen can be identified.
